# Comprehensive conservation profiling enables a robust multiplex RT-qPCR assay for simultaneous pan-detection and serotype discrimination of dengue virus

**DOI:** 10.1128/spectrum.00718-26

**Published:** 2026-09-02

**Authors:** Bohan Xu, Dongyan Xiong, Jinzhang Wang, Chunjing Zhao, Junhua Li, Hongping Wei, Junping Yu

**Affiliations:** 1 State Key Laboratory of Virology and Biosafety, Center for Biosafety Mega-Science, Wuhan Institute of Virology, Chinese Academy of Sciences 74614, Wuhan, China; 2 University of Chinese Academy of Sciences 74519, Beijing, China; 3 Centre for Novostics 627362 , Hong Kong, China; 4 Department of Chemical Pathology, Chinese University of Hong Kong, Prince of Wales Hospital 546484, Hong Kong, China; 5 Fujian Provincial Center for Disease Control and Prevention, Fujian Provincial Key Laboratory of Zoonosis Research 117864 , Fuzhou, China; Public Health Agency of Canada, Winnipeg, Manitoba, USA

**Keywords:** dengue virus, pan‐dengue, molecular diagnostics, multiplex real-time RT-qPCR

## Abstract

Dengue virus (DENV) remains a critical global health threat, necessitating rapid pan-detection and precise serotyping. In this study, we developed a multiplex real-time RT-qPCR assay for simultaneous detection and identification of DENV1–4. Guided by a systematic genomic conservation analysis of 4,754 sequences, we targeted the highly conserved 5′ untranslated region (5′ UTR) to design one pan-DENV and four serotype-specific primer-probe sets. The assay demonstrated robust analytical performance, with the limit of detection (LOD) ranging from 15.8 to 1.58 × 10^3^ copies/mL and the lowest LOD (15.8 copies/mL) observed for serotype I with specific primer-probe sets. There was no cross-reactivity with other common pathogens of the *Orthoflavivirus* or *Alphavirus* genus pathogens*,* and clinical specificity was further validated as 100% using a negative cohort of non-dengue febrile samples. Validation using 53 clinical DENV-positive samples showed 100% concordance with a reference diagnostic kit for serotype identification. While the pan-DENV set served as a high-specificity screening tool with broad reactivity, it exhibited a clinical sensitivity of 88.7%, particularly missing low-titer samples (Ct > 30). This discrepancy, coupled with the superior sensitivity of the serotype-specific sets, suggests the presence of incomplete genomic fragments in acute-phase sera, highlighting the advantage of our 5′ UTR-targeted approach in assessing viral genomic integrity. This dual-layered multiplex assay provides a robust, rapid, and cost-effective tool for clinical diagnosis and epidemiological surveillance.

**IMPORTANCE:**

Dengue fever is a leading cause of systemic viral disease worldwide. Co-circulation of four distinct serotypes complicates clinical management and increases severe disease risk. Here, we presented a novel multiplex RT-qPCR assay enabling simultaneous pan-DENV detection and serotype identification in two tubes, comprising six reactions including an internal control (IC), thereby streamlining dengue diagnosis. A key innovation is the systematic selection of the 5′ UTR as the diagnostic target, guided by big-data genomic analysis. Unlike assays targeting the 3′ UTR, which may overrepresent viral loads by capturing redundant subgenomic orthoflavivirus RNA (sfRNA), our 5′ UTR-targeted design provides a more stringent measure of genomic integrity. Furthermore, the modular performance of our assay combining broad screening via a pan-DENV set with high-sensitivity subtyping which offers a dual-layer diagnostic framework. This work provides a high-performance tool for timely clinical intervention and enhances our molecular understanding of DENV fragments in clinical specimens.

## INTRODUCTION

Dengue fever is a mosquito-borne infectious disease caused by the dengue virus (1). Belonging to the *Flaviviridae* family and *Orthoflavivirus* genus, the virus possesses a genome consisting of approximately 11 kb of single-stranded, positive-sense RNA, which encodes three structural proteins and seven non-structural proteins (2). The dengue virus exists as four antigenically distinct serotypes: dengue virus serotype 1 (DENV-1), serotype 2 (DENV-2), serotype 3 (DENV-3), and serotype 4 (DENV-4) (3). The primary vectors for dengue transmission are mosquitoes of the genus *Aedes*, particularly *Aedes aegypti* and *Aedes albopictus* (4–6). The larvae of these mosquitoes often breed in inappropriate water storage environments, thereby facilitating virus transmission. As these mosquito species are predominantly distributed in tropical and subtropical regions (2, 7, 8), and dengue outbreaks often exhibit seasonal patterns typically peaking during and after the rainy season, the incidence of dengue fever remains relatively high in these areas.

According to statistics from the World Health Organization (WHO), globally, from January 2024 to July 2025, there were 9,410,789 cumulatively confirmed dengue fever cases and 13,843 associated deaths (9). Currently, dengue fever is endemic in over 100 countries across Africa, the Americas, the Eastern Mediterranean, South-East Asia, and the Western Pacific regions. Among these, the Americas, South-East Asia, and the Western Pacific are the most severely affected, with Asia accounting for approximately 70% of global cases (10). Prior to 2000, dengue fever in China was primarily concentrated in the southern coastal and tropical regions (11). However, over time, the epidemic has gradually spread northward (12, 13). From January 2024 to July 2025, China reported a total of 25,587 confirmed cases (14). One modeling study predicts that approximately 390 million dengue virus infections occur globally each year, with about 96 million individuals manifesting clinical symptoms (10).

Currently, there are no effective specific treatments available for dengue fever or its severe forms (15). Symptoms in patients experiencing a secondary infection with a heterotypic dengue virus serotype typically do not diminish; instead, they may worsen due to the antibody-dependent enhancement (ADE) effect (15, 16). Antibodies generated during the primary infection may cross-react with the different serotype encountered in the secondary infection (17), thereby exacerbating disease severity and increasing the risk of progression to dengue hemorrhagic fever (DHF) and dengue shock syndrome (DSS) (18). The severity of dengue virus infection varies depending on factors such as serotype, genetic variations, and viral virulence (15, 19). Structural differences and variations in viral replication rates exist among different serotypes, which may differentially influence the pathogenesis of the disease (20, 21). Consequently, early detection and timely diagnosis of the infection type play a critical role in the effective management of dengue fever.

Common diagnostic methods for dengue fever include virus isolation, serological assays, and nucleic acid detection (3, 22). Virus isolation, a conventional and reliable diagnostic technique primarily achieved through cell culture, is limited by its requirement for sophisticated laboratory equipment and lengthy processing time (2, 23). Among serological assays, the IgM and IgG capture enzyme-linked immunosorbent assay (ELISA) is widely used. However, its effectiveness is constrained by cross-reactivity with other orthoflaviviruses and limited utility during periods of low antibody titers (24–26), such as the febrile viremic phase (27, 28). Furthermore, this method cannot effectively serotype the dengue virus (3). To complement these limitations, the detection of secreted Non-Structural Protein 1 (NS1) has emerged as another vital biomarker for early dengue diagnosis, and recent advancements, such as the development of monoclonal antibody pairs to distinguish the four serotypes in lateral flow assays, have further enhanced its rapid utility (29). Nevertheless, nucleic acid detection techniques, such as reverse transcription polymerase chain reaction (RT-PCR), can detect viral RNA during the early stage of the disease. Offering higher sensitivity and specificity, relative operational simplicity (30), rapid turnaround time, and lower cost, RT-PCR remains the preferred “gold standard” for dengue diagnosis and virus serotyping (23, 31). In current RT-PCR detection technologies for dengue virus detection and serotype identification, the commonly used approach involves combining specific primer-probe systems targeting the four serotypes to enable simultaneous detection (32). For example, in studies published by scholars such as Waggoner et al. in 2013, 2017, and 2018 (32–34), the pan-detection methods designed for the four serotypes of dengue virus did not employ a single primer-probe system to achieve detection across all serotypes. Instead, they combined primer-probe sets targeting specific serotypes to accomplish pan-detection of all four. While Songjaeng et al. in 2022 (35) proposed a pan-DENV detection system targeting the 3′ untranslated region (3′ UTR), their primer design was based on genomic data limited to Thailand and other Asian regions prior to December 2017, which may constrain its broad applicability against global DENV diversity. More importantly, targeting the 3′ UTR may introduce significant diagnostic bias due to the biological nature of DENV replication. During infection, the incomplete degradation of genomic RNA by host exonuclease XRN1 leads to the massive accumulation of subgenomic orthoflavivirus RNA (sfRNA), which corresponds to the 3′ UTR sequence (36). While the massive accumulation of sfRNA can result in artificially lower Ct values, potentially enhancing absolute detection sensitivity in degraded samples, it may not accurately reflect the concentration of functional, full-length viral genomes. In contrast, our assay strategically targets the 5′ untranslated region (5′-UTR), which offers several significant advantages over the 3′-UTR for pan-DENV detection. First, the 5′-UTR exhibits higher sequence conservation across all four serotypes due to the strong functional constraints on its secondary structures, such as stem-loops essential for viral replication and translation, thereby providing an ideal target for high-coverage primer design. Second, located at the very beginning of the genome, this region is less susceptible to the effects of partial RNA degradation during reverse transcription, as the initiation site for 5′−3′ degradation, the 5′-UTR does not yield stable subgenomic fragments like sfRNA, which contributes to improving the sensitivity and reliability of detection in clinical samples. Furthermore, its conserved regions are relatively concentrated, facilitating the design of a short-amplicon primer-probe system with high amplification efficiency and strong specificity, effectively reducing the risk of false negatives due to target region variation (32, 37, 38). Additionally, the specific detection primer-probes they employed were primarily based on earlier research findings (35). Lastly, while the article published by the research team of Luciani et al. (39) in 2024 also used a single primer-probe system to achieve pan-detection of the four dengue virus serotypes, their design similarly relied on existing literature and did not involve novel primer-probe development.

In this study, we leveraged a comprehensive analysis of high-quality complete genome sequences from all four DENV serotypes, retrieved from NCBI virus database up to 12 June 2025, to identify highly conserved genomic regions across all four serotypes. Using genome-wide multiple sequence alignment, we systematically evaluated conservation patterns across the viral genome and selected two functionally distinct targets based on their complementary strengths: the highly conserved 5′ untranslated region (5′ UTR) for pan-DENV detection, and the polyprotein gene, which harbors serotype-specific signatures despite overall functional conservation, for precise serotyping. Guided by this genome-wide conservation profiling, we designed a one-step real-time multiplex RT-qPCR system incorporating TaqMan probes and a human internal control. The system integrates a 5′ UTR-based pan-DENV primer-probe set for broad-spectrum sensitivity with polyprotein gene-based serotype-specific primer-probe sets, enabling simultaneous detection and differentiation of all four DENV serotypes in a streamlined workflow. This genomics-informed design ensures high analytical sensitivity, minimal cross-reactivity, and robust performance across diverse viral lineages, making it a powerful tool for rapid, reliable dengue diagnosis, surveillance, and outbreak response.

## MATERIALS AND METHODS

### Analysis of public dengue virus genomic data

To avoid analytical bias, 999 high-quality DENV sequences retrieved from the NCBI Virus database up to 12 June 2025 were selected for analysis. These sequences were chosen through balanced selection based on geographical location, host species, source of isolation, and genotyping criteria. The data set comprised 252 sequences for DENV-1, 251 for DENV-2, 250 for DENV-3, and 246 for DENV-4. Subsequently, conserved sequence analysis was performed on these 999 sequences following our previously published methodology (40). Reference sequences NC_001477.1 (DENV-1), NC_001474.2 (DENV-2), NC_001475.2 (DENV-3), and NC_002640.1 (DENV-4) were employed to identify conserved sequence fragments common to all four DENV serotypes as well as serotype-specific conserved fragments.

### Viruses

Dengue virus serotypes 1 to 4 were isolated from clinical samples of infected patients and subsequently amplified in *Aedes albopictus C6/36* cells (strain IVCAS9.087, National Virus Resource Center, Wuhan Institute of Virology, Chinese Academy of Sciences, Wuhan, China). The inactivated Zika virus, Japanese encephalitis virus, and Chikungunya virus were kindly provided by Prof. Bo Zhang in Wuhan Institute of Virology, Chinese Academy of Sciences, Wuhan, China.

### Nucleic acid extraction

A volume of 140 μL of viral sample (cell culture supernatant) was added to 560 μL of lysis buffer containing 1 μg/μL Carrier RNA (5.6 μL). RNA was then extracted using the QIAamp Viral RNA Mini kit (Qiagen) according to the manufacturer’s instructions. The extracted nucleic acids were stored at −80°C.

### Design of primers and probes

We designed primers and probes targeting the identified conserved sequence fragments using Beacon Designer (Version 8.13; Premier BioSoft, CA, USA) and Primer Premier 5.0 software. The six primer-probe sets are listed in Table 1. The FAM-labeled primer-probe set 1 which is modified by locked nucleic acid (LNA) targets the 5′-UTR region conserved across all four DENV serotypes, enabling pan-serotype detection of DENV. The FAM-channel primer-probe set 2 targets the polyprotein gene encoding the envelope protein E specific to DENV-1, facilitating DENV-1-specific detection. The HEX-labeled primer-probe set 3 targets the DENV-2-specific polyprotein gene for DENV-2 detection. The ROX-labeled primer-probe set 4 targets the DENV-3-specific polyprotein gene for DENV-3 detection. The HEX-labeled primer-probe set 5 targets the DENV-4-specific polyprotein gene for DENV-4 detection. The ROX-labeled primer-probe set 6 serves as an internal control targeting the RPP30 gene. Subsequently, the designed primer-probe sets were computationally analyzed against the downloaded full-length DENV sequences from the NCBI Virus database as of 12 June 2025. This *in silico* analysis comprised a total of 4,754 DENV sequences, encompassing 1,899 DENV-1 sequences, 1,643 DENV-2 sequences, 967 DENV-3 sequences, and 245 DENV-4 sequences. The purpose was to assess the match rate (coverage) of the five primer-probe sets—designed for pan-DENV detection and specific differentiation of DENV serotypes 1–4 against these downloaded full-length DENV sequences. Based on the analysis of self-dimer and cross-dimer formation using the Multiple Primer Analyzer tool (41), the primer-probe sets were assigned to two multiplex reactions as follows: Set 1 (DG-PAN-PP), Set 5 (DG4-SP-PP), and Set 6 (IC-PP) were combined in a single reaction tube for nucleic acid amplification, while Set 2 (DG1-SP-PP), Set 3 (DG2-SP-PP), and Set 4 (DG3-SP-PP) were combined in another single reaction tube for nucleic acid amplification.

**TABLE 1:** The sequences of the six primer-probe sets used in this study^*a*^

Set	Name	Sequences (5'−3')
Set1	DG-PAN-Forward	TWA**T**TAGAGAG**C**AGATCTC
	DG-PAN-Reverse	GTTG**A**CACRC**G**GTTTCTC
	DG-PAN-Probe	FAM-CGCGTTTCAGC**A**TA**T**TG**A**WAGRCG-BHQ1
Set2	DG1-SP-Forward	TGTGCATTGAAGCTAAAATATCA
	DG1-SP-Reverse	CGTCTTGTTCTTCCACCA
	DG1-SP-Probe	FAM-ACCACCACCGACTCAAGATGTCCAA-BHQ1
Set3	DG2-SP-Forward	CGAGAAATACGCCTTTCAATA
	DG2-SP-Reverse	CAGCATTCCAAGTGAGAATC
	DG2-SP-Probe	HEX-AACCGCGTGTCAACTGTGCAAC-BHQ2
Set4	DG3-SP-Forward	CAACCAACGGAAGAAGAC
	DG3-SP-Reverse	CGCCAACTGTGATCCAGT
	DG3-SP-Probe	ROX-AAACCGTCTATCAATATGCTGAAACGC-BHQ2
Set5	DG4-SP-Forward	GGTTGGTGAAGAGATTCTCA
	DG4-SP-Reverse	GTGGGATGGAAAGGACTC
	DG4-SP-Probe	HEX-AGCACCATCCGTAAGGGTCCT-BHQ1
Set6	IC-Forward	AGATTTGGACCTGCGAGCG
	IC-Reverse	GAGCGGCTGTCTCCACAAGT
	IC-Probe	ROX-TTCTGACCTGAAGGCTCTGCGCG-BHQ2

^*a*^ Bolded bases indicate nucleotides modified with locked nucleic acid (LNA).

### The procedure to determine DENV and serotypes by RT-qPCR assay

A RT-qPCR assay is prepared in two PCR tubes to determine whether an unknown sample is positive for DENV and, if positive, to identify the specific serotype. The RT-qPCR experiments were conducted using a BioRad CFX96 Deep Well Dx ORM RT-qPCR system (Bio-Rad Inc., Hercules, CA, USA). The polymerase used in the RT-qPCR process was HiScript II One Step qRT-PCR Probe Kit Q222 (Vazyme Biotech Co., Ltd, Nanjing, China), and the preparation of the detection mixture was carried out according to the manufacturer’s instructions. The RT-qPCR reaction mixture 1 was assembled as follows: 10 μL 2 × One step Q probe mix, 1 μL One step Q probe Enzyme mix, 0.4 μL 10 μM of each (DG-PAN-PP, DG4-SP-PP, IC-PP) Forward Primer, 0.4 μL 10 μM of each (DG-PAN-PP, DG4-SP-PP, IC-PP) Reverse Primer, 0.2 μL 10 μM of each (DG4-SP-PP, IC-PP) TaqMan Probe, 0.8 μL 10 μM of DG-PAN-PP TaqMan Probe, 5 μL Template DNA, and nuclease-free water to a final volume of 20 μL. The RT-qPCR reaction mixture 2 was assembled as follows: 10 μL 2 × One step Q probe mix, 1 μL One step Q probe Enzyme mix, 0.4 μL 10 μM of each (DG1-SP PP, DG2-SP-PP, DG3-SP-PP) Forward Primer, 0.4 μL 10 μM of each (DG1-SP-PP, DG2-SP-PP, DG3-SP-PP) Reverse Primer, 0.2 μL 10 μM of each (DG1-SP-PP, DG2-SP-PP, DG3-SP-PP) TaqMan Probe, 5 μL Template DNA, and nuclease-free water to a final volume of 20 μL. Six Ct values can be obtained for the six sets of primer-probes, labeled as Ct1, Ct2, Ct3, Ct4, Ct5, and Ct6 corresponding to the three channels, FAM, HEX, and ROX, respectively. Cycling conditions are as follows: 50°C for 15 min (RT step), 95°C for 30 s, 40 cycles of 95°C for 10 s, 55°C for 30 s.

The diagnostic system utilizes an integrated dual-tube parallel testing strategy. For each clinical specimen, dual-tube real-time PCR amplifications, comprising Tube 1 (DG-PAN-PP, DG4-SP-PP, and IC-PP) and Tube 2 (DG1-SP-PP, DG2-SP-PP, and DG3-SP-PP) are performed simultaneously to achieve both pan-DENV screening and serotype identification. A sample is defined as clinically positive for DENV if a specific amplification signal (Ct < 40) is detected in any of the reaction channels across the two tubes. This parallel configuration allows the high-sensitivity serotype-specific assays to act as a diagnostic backup for the universal PAN-DENV screen, particularly in specimens near the limit of detection (LOD).

### Determination of the limit of detection

The extracted viral nucleic acids of DENV-1 to DENV-4 were subjected to serial 10-fold dilutions (ranging from 10^−1^ to 10^−9^) in commercial Tris-EDTA buffer (TE buffer). These serially diluted nucleic acids at different concentrations were then used as templates for RT-qPCR. The lowest nucleic acid concentration detectable by RT-qPCR defined the limit of detection (LOD) for each primer-probe set targeting dengue virus. Furthermore, the results from RT-qPCR on the serially diluted dengue nucleic acids helped determine the appropriate nucleic acid template concentration for subsequent ddPCR analysis. Due to the absence of a ROX channel on the ddPCR instrument, the probe in Set 4 was conjugated with a FAM fluorophore for ddPCR. Droplet digital PCR (ddPCR) was subsequently performed using the Bio-Rad QX200 ddPCR system (Bio-Rad Inc., Hercules, CA, USA) to calculate the copy number of dengue virus nucleic acid at each dilution. Based on the tested concentrations of dengue virus nucleic acid and their corresponding measured copy numbers, the specific copy number corresponding to the LOD for each primer-probe set was calculated, thereby obtaining the precise copy number value at the exact LOD concentration. Diagnostic sensitivity, specificity, and their corresponding 95% confidence intervals (CIs) were calculated using the Clopper-Pearson exact method via GraphPad Prism 8.4.2.

### Cross-specificity and cross-detection tests

The specificity of the primer-probe sets designed in this study was evaluated by detecting genomic RNA from three viruses: Zika virus and Japanese encephalitis virus (both belonging to the *Orthoflavivirus* genus) and Chikungunya virus (belonging to the *Alphavirus* genus). Specificity testing was performed using both the Set 1 (DG-PAN-PP) primer-probe set and the four additional sets designed for differentiating dengue virus serotypes.

### Evaluation of clinical performance and specificity

To evaluate the diagnostic performance of the designed primer-probe sets and ensure their robustness against diverse viral lineages, a total of 73 clinical serum samples were utilized in this study. This cohort consisted of a DENV-positive validation group (*n* = 53) and a negative control group (*n* = 20).

The DENV-positive group, provided by the Fujian Provincial Center for Disease Control and Prevention, represents a diverse geographical and genetic snapshot of DENV. Specifically, the cohort included: international imported cases from nine countries across Southeast Asia, Africa, and the Middle East (Malaysia, Angola, Indonesia, Laos, United Arab Emirates [Dubai], Cambodia, Tanzania, the Philippines, and Thailand); domestic out-of-province cases from Yunnan and Guangdong, two major DENV-endemic regions in China; and intra-provincial cases distributed across seven major prefectures in Fujian (Fuzhou, Nanping, Quanzhou, Putian, Sanming, Xiamen, and Zhangzhou). Detailed information regarding the geographical origins of each sample is summarized in Supplemental material S1.

Clinical severity was classified according to WHO guidelines into dengue fever (DF) and dengue hemorrhagic fever (DHF). Serotype identification for all samples was independently confirmed using a reference kit (Dengue Virus Nucleic Acid Detection Kit, Model: DK0500M; Daan Gene, Guangzhou, China). The collection and use of these clinical samples were approved by the Medical Ethics Review Committee of the Fujian Provincial Center for Disease Control and Prevention.

To rigorously assess clinical specificity and rule out potential false-positives results, a negative control group was established consisting of 20 serum samples from patients with non-dengue febrile illnesses. These specimens were collected from individuals presenting with acute febrile symptoms clinically overlapping with early-stage dengue fever but were confirmed to be DENV negative using reference diagnostic methods. These patients presented with febrile symptoms clinically similar to early-stage dengue fever, providing a challenging and relevant negative cohort for differential diagnosis.

Viral RNA was isolated from all serum samples according to the respective manufacturers’ instructions. Specifically, the DENV-positive serum samples underwent RNA extraction using the TIANLONG Nucleic Acid Extraction Kit (Model: T324) following the manufacturer’s instructions. While the DENV-negative febrile specimens were processed using commercial viral RNA extraction kits from Vazyme Biotech Co., Ltd. (Nanjing, China) following the manufacturer’s instructions. The extracted RNA was stored at −80°C until use, and each sample was tested in duplicate for the RT-qPCR analysis to ensure technical reproducibility and diagnostic accuracy.

## RESULTS

### Quantification of dengue virus serotypes by multiplex real-time qPCR assay

**Fig 1:**
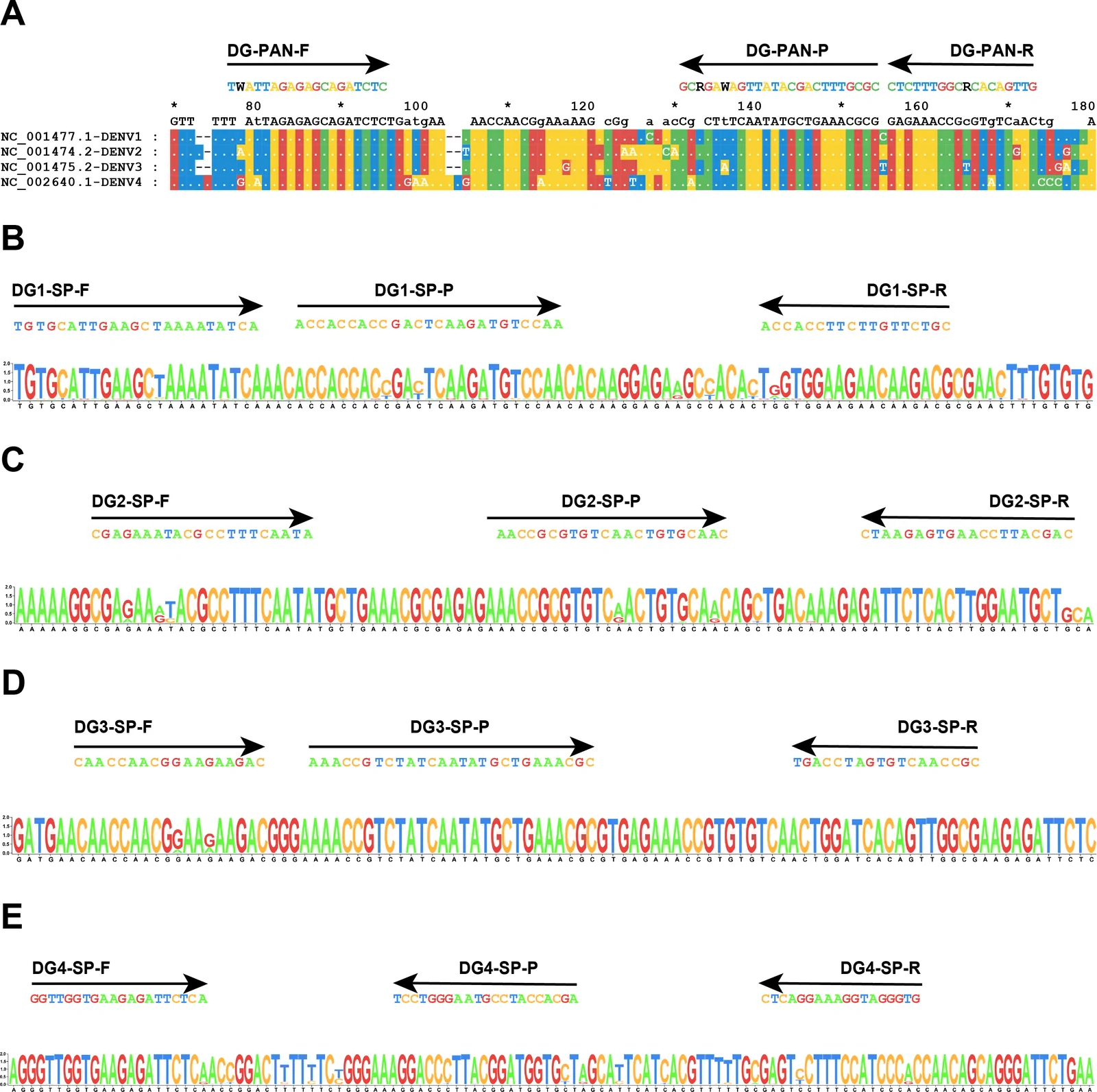
Schematic illustration of primer-probe set design targeting conserved genomic regions for pan-dengue and serotype-specific detection. (**A**) A universal primer-probe set for pan-DENV detection was designed to target a conserved region within the 5′ UTR region of the dengue virus genome. (**B–E**) Serotype-specific primer-probe sets were designed to target conserved regions in the polyprotein-coding gene unique to each of the four dengue virus serotypes (DENV-1 to DENV-4).

To validate the performance of the designed primer-probe sets, including the universal set for pan-dengue detection and the four serotype-specific sets, we utilized laboratory-cultured DENV 1–4 strains. Total RNA was extracted and served as standardized templates for assay validation. The testing results are summarized in Table 2. Additionally, the genomic locations of the amplicons generated by the five primer-probe sets (targeting conserved regions across the dengue genome) are illustrated in Fig. 1.

**TABLE 2:** Analytical specificity of the multiplex real-time RT-qPCR detection of dengue virus serotypes^*a*^

Pathogen	Strains (Serotype)	RT-qPCR protocols				
		DG-PAN-PP	DG1-SP-PP	DG2-SP-PP	DG3-SP-PP	DG4-SP-PP
DENV	DENV1	24.13	19.62	NA	NA	NA
	DENV2	13.95	NA	15.02	NA	NA
	DENV3	14.43	NA	NA	16.56	NA
	DENV4	18.40	NA	NA	NA	19.47

^*a*^ "NA" means samples with no amplification.

### Analytical sensitivity of the multiplex real-time qPCR assay

The sensitivity of the developed RT-qPCR assay was evaluated using pre-quantified RNA samples of DENV-1 to DENV-4. Nucleic acids from each serotype were serially diluted 10-fold with TE buffer and tested via RT-qPCR. The limits of detection (LOD) were determined as follows: Table 3 also provides an overall summary.

**TABLE 3:** Summary of LOD for each primer-probe set used in this study

Strains (Serotype)	LOD (copies/mL)				
	DG-PAN-PP	DG1-SP-PP	DG2-SP-PP	DG3-SP-PP	DG4-SP-PP
DENV1	1.58 × 10^3^	1.58 × 10¹	/^*a*^	/	/
DENV2	2.8 × 10^2^	/	2.8 × 10^2^	/	/
DENV3	2.12 × 10¹	/	/	2.12 × 10^2^	/
DENV4	3.88 × 10^2^	/	/	/	3.88 × 10^2^

^*a*^ "/” indicates that no LOD values were determined for those primer-probe sets against the corresponding dengue virus serotypes.

For the universal primer-probe set (Set 1), the LODs in copy numbers were:

DENV-1: 1.58 × 10^3^ copies/mL

DENV-2: 2.8 × 10^2^ copies/mL

DENV-3: 2.12 × 10^1^ copies/mL

DENV-4: 3.88 × 10^2^ copies/mL

For the serotype-specific primer-probe sets, the LODs in copy numbers were:

DENV-1 (Set 2): 1.58 × 10^1^ copies/mL

DENV-2 (Set 3): 2.8 × 10^2^ copies/mL

DENV-3 (Set 4): 2.12 × 10^2^ copies/mL

**Fig 2:**
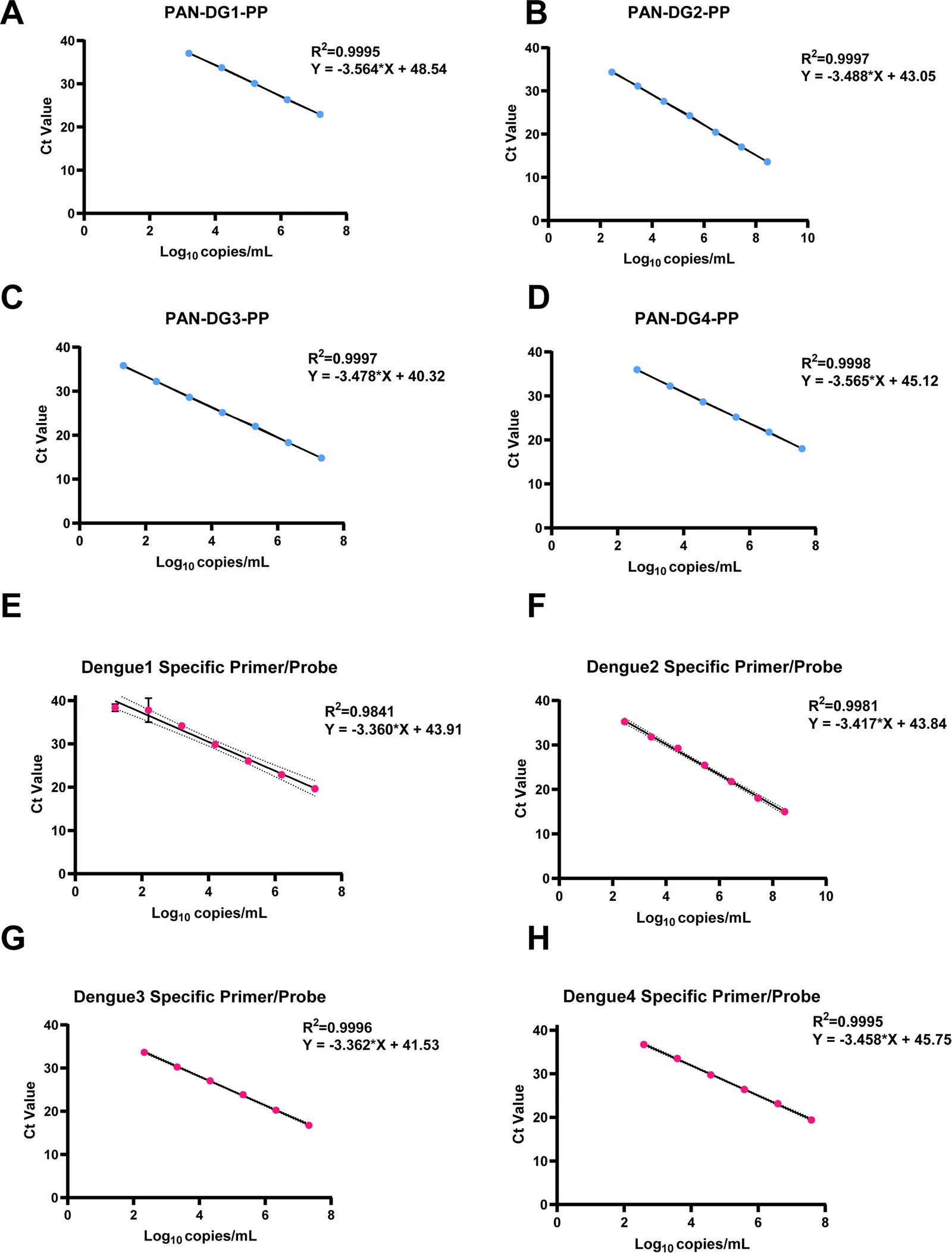
Standard curves and amplification efficiencies of the DG-PAN-PP (**A–D**) and four serotype-specific primer-probe sets (**E–H**). The DG-PAN-PP assay exhibited amplification efficiencies of 90.80%, 93.51%, 93.87%, and 90.77% for DENV-1, DENV-2, DENV-3, and DENV-4, respectively. The serotype-specific assays showed efficiencies of 98.44% (DG1-SP-PP), 94.13% (DG2-SP-PP), 98.35% (DG3-SP-PP), and 94.62% (DG4-SP-PP). Error bars indicate standard deviations derived from duplicate measurements.

DENV-4 (Set 5): 3.88 × 10^2^ copies/mL. Standard curves plotting Ct values against log_10_-transformed template copy concentration for each primer-probe set are presented in Fig. 2. The amplification efficiencies of the DG-PAN-PP primer-probe set for DG1, DG2, DG3, and DG4 reached 90.80%, 93.51%, 93.87%, and 90.77%, respectively, demonstrating its high amplification efficiency and promising potential for experimental application as a universal detection system for dengue virus. For the serotype-specific assays, the amplification efficiency of DG1-SP-PP for DG1 was 98.44%, DG2-SP-PP for DG2 was 94.13%, DG3-SP-PP for DG3 was 98.35%, and DG4-SP-PP for DG4 was 94.62%. These results indicate that all four serotype-specific primer-probe sets exhibit high amplification efficiency and good specificity for their respective dengue virus serotype target sequences. Given the potential for interference phenomena such as primer dimers between primer pairs, to ensure the accuracy of the detection, we have grouped the aforementioned five sets of primers and probes into two sets for testing: one set comprising DG-PAN-PP, DG4-SP-PP, and IC-PP, and the other set including DG1-SP-PP, DG2-SP-PP, and DG3-SP-PP.

### Cross-specificity and cross-detection tests

The specificity of the primer-probe sets used in this study was evaluated through cross-reactivity tests against RNA from other relevant viruses. As shown in Table 4, no cross-reactivity was observed with RNA from Zika virus and Japanese encephalitis virus (both belonging to the genus *Orthoflavivirus*), as well as Chikungunya virus (genus *Alphavirus*). These findings confirm that the primer-probe sets employed in the developed method exhibit high specificity for dengue virus detection.

**TABLE 4:** Cross-reactivity of dengue virus primer-probe sets against non-target orthoflaviviruses and alphaviruses^*a*^

Pathogen	RT-qPCR protocols				
	DG-PAN-PP	DG1-SP-PP	DG2-SP-PP	DG3-SP-PP	DG4-SP-PP
Zika	NA	NA	NA	NA	NA
JEV	NA	NA	NA	NA	NA
CHIKV	NA	NA	NA	NA	NA

^*a*^ "NA" means samples with no amplification.

### Performance evaluation and clinical specificity using clinical serum samples

To evaluate the diagnostic performance and clinical specificity of the designed primer-probe sets, we tested a total cohort of 73 clinical serum samples, including 53 DENV-positive specimens and 20 DENV-negative specimens.

**Fig 3:**
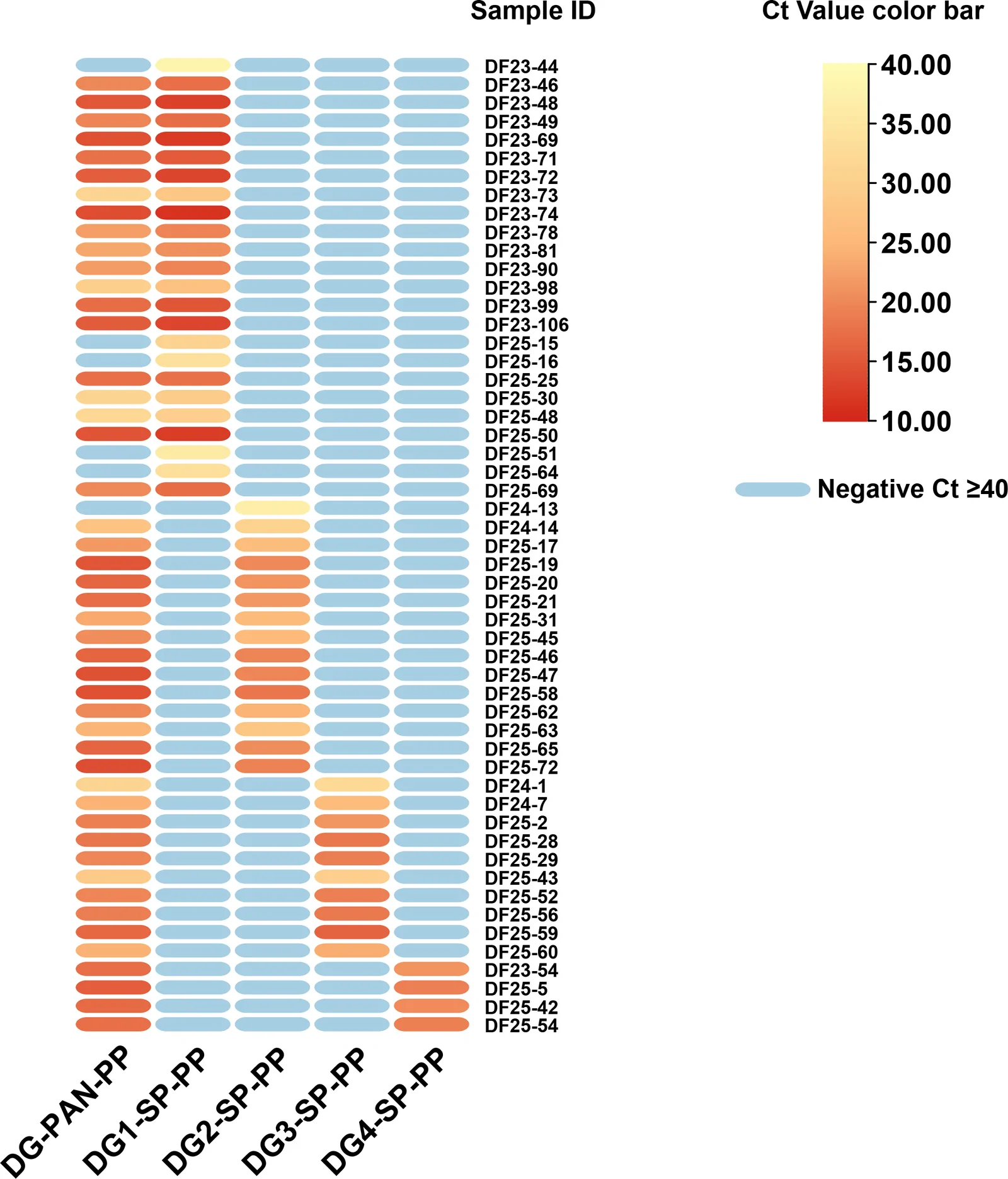
Performance evaluation of the pan-DENV and serotype-specific primer-probe sets using 53 DENV-positive clinical samples. The heatmap displays the Ct values obtained with the universal (pan-DENV) assay and the four serotype-specific RT-qPCR assays (DENV-1 to DENV-4) across the clinical cohort. Each row represents an individual clinical sample, and each column corresponds to one of the five assays. The color scale (right) depicts Ct values ranging from 10 (dark red) to 40 (light yellow). Ct values ≥ 40 (light blue) are displayed in uniform light blue and were interpreted as negative for DENV.

A heatmap was constructed using the Ct values obtained from 53 DENV-positive clinical samples. Samples were categorized into four groups (DENV1–4) based on the results of a reference diagnostic kit. For data visualization, any sample with no amplification or a Ct value ≥40 was assigned a value of 40. The heatmap was generated using TBtools-II (v2.400). To emphasize the serotype-specific detection pattern, samples were manually sorted by their clinical serotypes rather than using unsupervised hierarchical clustering. The color gradient represents the Ct values, with dark red indicating high viral loads (lower Ct values) and light blue representing negative results (Ct ≥ 40). As shown in Fig. 3, a distinct diagonal pattern was observed for the four specific primer sets, confirming zero cross-reactivity among the different DENV serotypes even when testing diverse strains from different global origins.

The clinical performance of the PAN primer-probe set was validated concurrently using these 53 positive samples. The PAN set successfully identified the majority of samples across all four serotypes. However, a discrepancy was observed in 6 samples that exhibited high Ct values (Ct > 30) in the reference diagnostic kit, where the PAN set failed to yield a detectable signal, resulting in an overall clinical sensitivity of 88.7% (95% CI: 77.0%–95.7%). In contrast, the serotype-specific assays correctly typed all positive specimens, achieving a sensitivity of 100% (95%CI: 93.3%–100%).

This localized reduction in sensitivity at lower viral loads underscores the critical importance of our dual-tube strategy. In cases of ultra-low viremia, specialized serotype-specific primers can occasionally exhibit a marginal sensitivity advantage over universal targets due to optimized binding kinetics for their exact sequence match. Rather than indicating a limitation of the individual components, these results demonstrate the complementary nature of our system: the serotype-specific sets serve as a vital diagnostic backup, ensuring that even specimens with minimal viral loads, which might escape a universal screen are successfully captured and typed.

From a biological perspective, this sensitivity variance may also be attributed to the genomic characteristics of DENV during acute infection. Previous studies have indicated that a significant proportion of "incomplete" or "defective" DENV genomic fragments circulate in the sera of acute-phase patients (35). In samples with low viral titers, the stochastic distribution or degradation of specific target sequences in these fragmented genomes may disproportionately affect the PAN-DENV set. Thus, while the PAN set provides a robust primary screen, its performance at the LOD is a reflection of both primer dynamics and the genomic integrity of the viral RNA in clinical specimens.

For 20 DENV-negative specimens, no amplification signals were detected (Ct ≥ 40 or NA) in any of the negative samples for either the pan-DENV detection set or the four serotype-specific sets (Table 5), demonstrating 100% clinical specificity (20/20) (95%CI: 83.2%–100%).

**TABLE 5:** Clinical specificity of the multiplex RT-qPCR assay against non-dengue febrile samples

Sample group	No. of samples	RT-qPCR protocols				
		DG-PAN-PP	DG1-SP-PP	DG2-SP-PP	DG3-SP-PP	DG4-SP-PP
Total	20	0/20(Neg)	0/20(Neg)	0/20(Neg)	0/20(Neg)	0/20(Neg)
Clinical Specificity	–^*a*^	100%	100%	100%	100%	100%

^*a*^ –, not applicable.

## DISCUSSION

Dengue fever remains a major global public health challenge, with all four DENV serotypes co-circulating in over 100 tropical and subtropical countries (42, 43). In the absence of effective antiviral therapies or a universally protective vaccine (44), early and accurate diagnosis is particularly critical not only for timely clinical management but also for mitigating the heightened risk of severe disease during secondary heterologous infections, which are strongly associated with dengue hemorrhagic fever (DHF) and dengue shock syndrome (DSS) (18).

Serological assays detecting DENV-specific IgM and IgG antibodies are widely used but suffer from delayed seroconversion (typically 7–8 days post-symptom onset) and cross-reactivity with other orthoflaviviruses, limiting their utility in the acute diagnostic window (18). Molecular approaches like RT-qPCR offer a more reliable alternative for early diagnosis. However, many existing assays either lack serotype resolution or exhibit uneven sensitivity across serotypes (2, 45, 46), underscoring the need for a balanced diagnostic strategy that combines broad detection coverage with high resolution serotyping.

To address this gap, we developed a dual-tube multiplex RT-qPCR assay that simultaneously achieves pan-DENV detection and precise serotyping. This design leverages two rationally selected genomic targets: The 5′ UTR, chosen for pan-detection due to its exceptional sequence conservation, stable secondary structure, and position at the 5′ terminus, making it less susceptible to RNA degradation and, thus, ideal for robust detection across all four serotypes; the polyprotein gene, selected for serotyping based on its functional constraints and serotype-defining variation, enabling precise discrimination despite high overall conservation. The dual-target strategy achieved both high analytical specificity (down to 15.8 copies/mL for serotype 1-specific detection) and excellent specificity, with no cross-reactivity against Zika virus, Japanese encephalitis virus, or Chikungunya virus (Tables 3 and 4). Importantly, *in silico* analysis confirmed near-complete compatibility of all primer-probe sets with 4,754 global DENV sequences deposited in the NCBI Virus database as of 12 June 2025 (Supplemental material S2 including Tables S1 to S13, displaying the matching analyses between the five primer-probe sets and indicating high matching rates of 94.97%, 100%, 100%, 100%, and 99.59% for DG-PAN-PP, DG1-SP-PP, DG2-SP-PP, DG3-SP-PP, and DG4-SP-PP, respectively), supporting the assay’s potential applicability across diverse endemic regions and future viral variants.

A key innovation of our approach lies in the deliberate prioritization of the 5′ UTR over the conventionally targeted 3′ UTR as the universal screening marker (35). Mechanistically, host exonuclease XRN1-mediated incomplete viral RNA decay yields an abundance of non-translatable subgenomic orthoflavivirus RNA (sfRNA) corresponding to the 3′ UTR. While this subgenomic accumulation provides a natural signal amplification effect that enhances clinical sensitivity in degraded or late-presentation specimens, 3′ UTR-targeted assays risk inflating viral load estimates and obscuring true genomic integrity. In contrast, our comparative study demonstrated that the 5′ UTR set consistently yielded lower Ct values (∆Ct 0.5–3.0) (Supplemental material S3). This performance advantage likely results from reduced interference from complex, enzyme-inhibiting secondary structures inherent in the 3′ UTR. Furthermore, the 5′ UTR possesses a significantly higher degree of sequence conservation across diverse orthoflavivirus lineages, effectively minimizing primer-probe mismatches caused by the high mutational possibility and evolutionary divergence characteristic of the 3′ UTR. From a structural perspective, while the 5′ UTR interacts with the 3′ UTR to form a closed-loop initiation complex (37), the 5′ UTR remains under evolutionary constraints to maintain sufficient conformational flexibility for host ribosomal scanning and assembly. By targeting the translation initiation site within the 5′ UTR, our assay demonstrates superior detection performance, providing a more conservative, biologically accurate representation of the intact, functional viral genome.

The clinical evaluation of our dual-assay framework revealed a nuanced trade-off between universality and absolute sensitivity. Notably, the pan-DENV assay failed to detect six low-titer clinical specimens (Ct > 30) that were successfully identified by the serotype-specific primers. This discrepancy reflects the thermodynamic cost of incorporating degenerate bases into universal primers to accommodate inter-serotype variations, which slightly reduces binding efficiency compared to the highly optimized, serotype-specific polyprotein primers. The PAN set provides rapid, broad screening, while the serotype-specific components deliver high-sensitivity confirmation and precise typing. The clinical robustness of this dual-layered architecture is further reinforced by its diagnostic accuracy; our evaluation of a negative cohort consisting of 20 specimens from non-dengue febrile illnesses yielded 100% specificity with zero false-positive signals, confirming reliable differential diagnosis despite overlapping clinical presentations.

Due to the fluorescence channel limitations of most common real-time PCR instrumentation (typically ≤ 5 channels), the six-target system (PAN, DENV1-4, and IC) was partitioned into a dual-tube format. IC is present in the first tube, serving as a proxy for the template quality and reaction environment of the parallel second tube, as both reactions utilize the same RNA extract and thermal conditions. This strategy ensures maximum compatibility with existing laboratory infrastructure while maintaining robust diagnostic oversight. Future work will focus on incorporating spectrally distinct fluorophores to enable true single-tube multiplexing. Such consolidation would not only simplify the workflow but also conserve precious clinical material and potentially enhance sensitivity by allowing a larger sample input volume.

While our current cohort (*n* = 73) is modest, its broad genetic and geographical diversity, spanning imported cases from nine countries and domestic samples from endemic Chinese provinces, provides robust epidemiological validation. Moving forward, large-scale, multi-center studies will be essential to further verify the assay’s predictive value in broader populations. Furthermore, given the overlapping symptoms and distributions of arboviruses like ZIKV, JEV, and CHIKV, our PAN-DENV design (utilizing a single primer-probe set) offers a distinct advantage by conserving multiplexing capacity that can be readily allocated to other pathogens. This allows for the seamless integration of additional primer-probe sets into a single reaction, creating a comprehensive syndromic panel. Such an expanded system would enable the simultaneous detection of multiple acute febrile pathogens, significantly enhancing clinical decision-making in regions where co-circulation is prevalent.

### Conclusion

In conclusion, we have successfully established and validated a multiplex RT-qPCR assay for simultaneous pan-detection and serotyping of all four DENV serotypes. Guided by genome-wide conservation analysis of global sequences, we targeted the highly conserved 5′ UTR for universal detection, ensuring broad reactivity while avoiding subgenomic RNA artifacts associated with 3′ UTR-based assays, and serotype-specific regions within polyprotein gene for precise typing. Our clinical data demonstrate that the assay is highly robust for routine screening and provides critical serotyping information essential for outbreak monitoring. Moreover, the observed performance in low-titer samples underscores the biological complexity of viral RNA in acute infections and validates our 5′ UTR-targeted approach as a biologically relevant diagnostic strategy. This dual-tube assay represents a significant advancement in DENV molecular diagnostics, offering a scalable solution for both clinical laboratories and public health surveillance systems in endemic regions.

## Data Availability

Data will be made available on request.

## SUPPLEMENTAL MATERIAL

Supplemental material S1 spectrum.00718-26-s0001.xlsx A detailed overview of the 53 clinical dengue virus samples analyzed in this study.

Supplemental material S2 spectrum.00718-26-s0002.xlsx Tables S1 to S13, showing the matching analysis between the primer-probe sets and dengue virus genome sequences.

Supplemental material S3 spectrum.00718-26-s0003.docx Preliminary comparative evaluation of 5′-UTR and 3′-UTR target platforms.

ASM does not own the copyrights to Supplemental Material that may be linked to, or accessed through, an article. The authors have granted ASM a non-exclusive, world-wide license to publish the Supplemental Material files. Please contact the corresponding author directly for reuse.
