# Supplementary material for: Comprehensive conservation profiling enables a robust multiplex RT-qPCR assay for simultaneous pan-detection and serotype discrimination of dengue virus
Source: Microbiol Spectr. 2026 Sep 2;14(10):e00718-26. doi: 10.1128/spectrum.00718-26 (PMC13689395; doi:10.1128/spectrum.00718-26)
Supplement: Supplementary file 1 [file spectrum.00718-26-s0003.docx]

**Supplementary Material S3: Preliminary Comparative Evaluation of 5' UTR and 3' UTR Target Platforms**

**1. Rationale and Objective**

The selection of an optimal genomic target is critical for balancing the multi-serotype universality and amplification efficiency of pan-DENV screening assays. While the 3' untranslated region (3' UTR) has been conventionally utilized in several expanded molecular diagnostic designs, it is known to generate abundant subgenomic orthoflavivirus RNA (sfRNA) degradation artifacts during active replication, which may distort clinical quantification ^1^. To empirically validate the selection of the 5' UTR as the primary screening target for our dual-tube framework, a preliminary head-to-head performance comparison was conducted against a previously validated reference pan-DENV assay targeting the 3' UTR ^2^. This evaluation aimed to benchmark baseline amplification efficiencies and template accessibility across all four globally circulating DENV serotypes.

**2. Methodological Approach**

Standardized viral RNA extracts from DENV-1, DENV-2, DENV-3, and DENV-4 were subjected to parallel testing using identical master mix (Vazyme Q222, Vazyme Biotech, China) and real-time platforms (Bio-Rad CFX96 System) to eliminate confounding assay variables. Ten-fold serial dilutions representing distinct viral load, ranging from high-titer initial concentrations to low-copy thresholds were evaluated in triplicate. Each primer-probe system was executed under its respective thermocycling profiles optimized for maximum binding kinetics. Diagnostic performance was quantified directly via the resulting threshold cycle (Ct values) obtained at identical genomic template inputs.

**3. Empirical Findings and Target Justification**

The comparative amplification profiles are illustrated in Figure S1. Across all four serotypes and at every tested concentration gradient, the 5' UTR-targeted primer-probe set consistently out-performed the 3' UTR reference framework.

The 5' UTR set demonstrated uniformly accelerated amplification kinetics, yielding earlier signals by approximately 0.5 to 3.0 cycles compared to the 3' UTR platform.

This systemic reduction in Ct values provides strong empirical evidence of superior primer-template thermodynamic affinity and enhanced enzymatic processivity within the 5' UTR region. These features effectively overcome the hindrance often caused by the complex, rigid secondary structures inherent in the 3' UTR. Consequently, these data support the inclusion of the 5' UTR as the core universal screening target in our assay, mitigating the risk of low-viremia diagnostic escape without relying on misleading non-infectious subgenomic fragments.

Figure S1. Comparison of amplification efficiency between the 5'-UTR and 3'-UTR targeted pan-DENV primer-probe sets. Serial dilutions of RNA templates from all four DENV serotypes (DENV-1 to DENV-4) were evaluated in parallel. The pink bars represent the Ct values obtained using the 5'-UTR-targeted pan-DENV assay developed in this study, while the blue bars represent the performance of the reference 3'-UTR-targeted universal assay. Data are presented as the mean ± standard deviation (SD) derived from three independent replicates (n=3) for each concentration gradient. The uniformly lower Ct values of the 5'-UTR set across all viral loads indicate its superior analytical sensitivity and enhanced amplification performance.

**References**

1	Niu, X. *et al.* Pseudoknot length modulates the folding, conformational dynamics, and robustness of Xrn1 resistance of flaviviral xrRNAs. *Nature communications* **12**, 6417, doi:10.1038/s41467-021-26616-x (2021).

2	Songjaeng, A. *et al.* Development of a Singleplex Real-Time Reverse Transcriptase PCR Assay for Pan-Dengue Virus Detection and Quantification. *Viruses* **14**, doi:10.3390/v14061271 (2022).
